# Supplementary material for: Inter-lower limb and intra-lower limb muscle activity correlations during walking: A comparative study of stroke patients and healthy individuals
Source: PLoS One. 2025 May 12;20(5):e0319955. doi: 10.1371/journal.pone.0319955 (PMC12068577; doi:10.1371/journal.pone.0319955)
Supplement: Attachment — Submitted filename: PONE-D-24-38957_review_S_Angelova.docx [file pone.0319955.s001.docx]

Review

Title of paper: Lower limb muscle activity during walking: A comparative study of stroke patients and healthy individuals

With authors: Thanita Sanghan, Nusreena Hohsoh, Goran Stojanović, Rezaul Begg,

Surapong Chatpun

General comments:

1. In the title it is better to accent the inter-limb and intra-limb correlations, that you investigate. It is not just about muscle activity.
2. Row 20 - By examing the root mean square (RMS) values and the area under the curve of RMS envelope of EMG for the gluteus medius (GM), rectus femoris (RF), biceps femoris (BF), medial gastrocnemius (MG), and peroneus longus (PL), this research was designed to enhance our understanding of muscle coordination and its complications in stroke gait rehabilitation– specify that GM, RF, BF, MG, PL are muscles.
3. Row 25 - Stroke patients exhibited knee hyperextension, attributed to muscle weaken and compensatory mechanisms, contributing to abnormal gait patterns such as stiff knee gait. Healthy participants showed muscle weakness predominantly in the proximal muscles. – Where is the difference? RF is responsible for knee extension and is a proximal muscle in your study (for stroke patients). The two proximal muscles here are GM and RF (for healthy people). A stiff knee gait could result from spasticity, not muscle weakness.
4. Row 35 - The findings highlight the necessity of focusing on training distal muscles in stroke patients or core/proximal muscles in cases where the the ankle joint is rarely moved. – If the weaknesses are found in the proximal muscles, why people should train distal?
5. In the Introduction is missing a review of other scientists work in the field, for example: https://www.arquivosdeneuropsiquiatria.org/article/muscle-activity-during-gait-following-stroke/; https://www.sciencedirect.com/science/article/abs/pii/S1388245720303436; https://pmc.ncbi.nlm.nih.gov/articles/PMC7028468/; <https://www.nature.com/articles/s41597-021-008813>; <https://pmc.ncbi.nlm.nih.gov/articles/PMC7502709/>

etc…

6. You need clearly to state the purpose of the study before the Methods section.
7. Row 66 - The participants were fifteen stroke patients and fifteen healthy individuals recruited from Songklanagarind hospital from 30 November 2021 to 14 August 2022 – Were both group participants recruited from the hospital?
8. Row 69 - Stroke patients with any history of musculoskeletal problems, other neurological conditions, lower limb arthroplasty, major falls, and fear of falling were excluded.- This sentence oposes of the mentioned previous- Stroke patients exhibited knee hyperextension, attributed to muscle weaken and compensatory mechanisms, contributing to abnormal gait patterns such as stiff knee gait. Knee hyperextension, weak muscles, compensatory mechanisms, abnormal gait patterns, and stiff knee gait are all neurological and/or musculoskeletal problems.
9. In Table 1 you give information about the affected side of the stroke patients. Did you assess the dexterity of all participants? It is important because you find further dissimilarities in healthy subjects too. At what recovery stage of [Brunnstrom](https://www.google.com/url?sa=t&source=web&rct=j&opi=89978449&url=https://www.physio-pedia.com/Brunnstrom_Movement_Therapy&ved=2ahUKEwin9sjGzqeJAxV3efEDHTkhCsUQFnoECBQQAQ&usg=AOvVaw3SS71sdwm73oO1wm9SkWsa)

are the hemiparetic subjects – this gives reflection to the results. Have you assessed some of the following: NIHSS, Fugl-Meyer, Glasgow Coma Scale, Hunt scale, Hess scale, Barthel Index, and Functional Independence Measure?

10. Row 81 – remove natural – it is acquired for stroke patients.
11. You can give a picture of such investigated stroke patients during examination. Better to see the attached devices and the walkway.
12. How did you choose the muscles to investigate? Do you use some worldwide recommendations for EMG examinations like the Seniam protocol?
13. Row 96 - The collected EMG data underwent filtering using a 30-450 Hz bandpass filter and a 50 Hz notch filter. - How did you choose that frequency range?
14. Besides Spearman it is good to mention other methods for estimating the surface EMG activity of humans like: <https://www.mdpi.com/2297-8747/29/1/8>
15. Row 146 - In the stroke patient group, significant differences between the affected and non-affected sides in the RMS values were found in… - You find significant differences in RMS values between the left and right sides in healthy people, which is expected due to dexterity. So here you should compare the left and right limbs in the right affected and separately in left affected stroke patients. Furthermore, it is important to say if all participants are right-handed, left-handed, or ambidextered. In your research ‘affected side ‘ includes as right, as well as the left arm. Such a comparison is unacceptable. Separate the results according to the affected side (R/L) 6/9.
16. Figs 2,3,4, and 5 are blurred. Is that real-time gait cycle presented? Is that mean, that the phases of all participants are equal-timed?
17. Specify in the text of figures 2,3,4, and 5 which one is for the right and which is for the left limb.
18. Specify in the text of figures 6 - a, b, c, d, and 7 - a, b, c, d, what information brings. It is unclear. You do not have a presentation of the figures in the text similar to 8 and 9 in row 227.
19. Row 247 – You talk about the ipsilateral (left) and contralateral (right) sides in healthy group. Termins ipsilateral and contralateral marks the body side in some kind of reference to lateral lesions. They are normally used for stroke patients. How did you decide to call the left leg ipsilateral and the right contralateral?
20. Row 288 - safe and effective movement throughout this phase, the current study identified that the RF is crucial in controlling knee extension and stabilizing the knee joint [14]. - You do not examine other knee extensors, so you can not say that.
21. Row 302 The GM, primarily involved in hip abduction, also functions as a secondary hip rotator, crucial for stabilizing the hip and assisting the BF in controlling thigh movement for hip extension during stance. - secondary hip **internal** rotator.
22. Row 314 In this study, the BF exhibits a dominant function facilitating knee flexion. – Again - dominant function among which knee extensors? In this study, you investigate just BF as a knee extensor!
23. Row 346 Likewise, Lim et al. found that individuals with distal muscle weakness experienced greater instability during forward and backward perturbations, whereas those with proximal muscle weakness were only unstable during backward perturbations [24]. - Citation number 24 is Horlings CGC, Küng UM, van Engelen BGM, Voermans NC, Hengstman GJD, van der Kooi AJ, et al. Balance control in patients with distal versus proximal muscle weakness. Neuroscience.479 2009;164(4):1876-86. not Lim et al. No existence of not Lim et al. at all. Please revise the references.
24. Row 362. No activity in the BF was observed due to stroke patients being unable to perform knee flexion. On the non-affected side, activity was observed in all six muscles. These findings are consistent with Chow et al., [27] which suggested a co-contraction of the TA, MG, and PL, typically antagonist muscles that ensure smooth ankle motion. – No activity in the BF? Being unable to perform knee extension does not mean, that no muscle action is registered. Sometimes there is muscle activation, but there is not enough power to move that limb. Does that refer to both limbs? This sentence needs to be changed. You talk about **BF** activity and after that say this correlates to Chow‘s findings about co-contraction of the **TA, MG, and PL**. Maybe a mistake appeared.
25. Row 366 Our study suggests a potential therapeutic approach based on age-related weakness in proximal muscles among healthy individuals, which could heighten the risk of falls when distal muscles are intensively used, leading to increased local fatigue – Therapy is for disabled and ill patients, training is for healthy subjects.
26. Row 387 Stroke patients exhibited knee hyperextension, often resulting from muscle weakness and compensatory strategies, contributing to abnormal coordination patterns such as stiff knee gait – In <https://pubmed.ncbi.nlm.nih.gov/26522901/> knee hyperextension is related to motor, sensory, and proprioceptive loss; spasticity; and/or ataxia on hemiparetic gait, not muscle weakness. Show your source.
