# Supplementary material for: Inter-lower limb and intra-lower limb muscle activity correlations during walking: A comparative study of stroke patients and healthy individuals
Source: PLoS One. 2025 May 12;20(5):e0319955. doi: 10.1371/journal.pone.0319955 (PMC12068577; doi:10.1371/journal.pone.0319955)
Supplement: Attachment — Submitted filename: Response to Reviewer_PONE_submitted.docx [file pone.0319955.s005.docx]

Responses to reviewers

**Reviewer 1**

1. Line 22: The paper lists muscles but omits the tibialis anterior (TA).

**Response:** Thank you for your point out. We have added “tibialis anterior (TA) in the abstract already as shown below.

This study investigated muscle activity, intra-, and inter-muscular correlations during gait phases, including first double support (DS1), single support (SS), second double support (DS2), and swing (SW) in both healthy individuals and stroke patients. By examining the root mean square (RMS) values and the area under the curve of RMS envelope of EMG for the gluteus medius (GM), rectus femoris (RF), biceps femoris (BF), medial gastrocnemius (MG), tibialis anterior (TA) and peroneus longus (PL) muscles, this research was designed to enhance our understanding of muscle coordination and its complications in stroke gait rehabilitation.

2. Several references cited in the discussion are not introduced in the introduction, which disrupts the flow of the manuscript and the background context.

**Response:** Thank you for this comment. We have rewritten the manuscript in the introduction and added some references mentioned earlier in the discussion as shown below.

In healthy individuals, walking requires the coordinated effort of various muscles to ensure smooth and efficient movement through the different phases of the gait cycle. In contrast, stroke patients typically reveal asymmetry in muscle activity and coordination between the affected and non-affected limbs [7] and the analysis of muscle synergies is a vital tool for assessing motor impairment and evaluating the effectiveness of rehabilitation [8]. Gait impairments in stroke patients are typically characterized by asymmetry and slow walking speed, often due to motor weakness, motor control deficit, sensory loss, proprioceptive issues, or ataxia [9, 10]. In addition, muscle dysfunction contributes to gait problems such as stiff knee gait, knee hyperextension, and foot drop. These impairment combine with compensatory strategies like circumduction or vaulting, further disrupt foot clearance and reduce walking efficiency [11].

3. It is not specified how the EMG sensors were placed. Did you follow some guidelines? Providing this information would enhance the repeatability of the study.

**Response:** Thank you for your comment. For the protocol of EMG attachment, we followed the SENIAM protocol which provides guidelines for standardized electrode placement, ensuring consistency and comparability in EMG measurement. Therefore, we have additionally added the SENIAM protocol in the texts for EMG examination as shown below.

Subsequently, Ag/AgCl electrodes were placed at the motor point of the targeted muscles, following the specific guidelines outlined in the SENIAM protocol.

4. The type and order of filters used to process EMG signals are not detailed. Additionally, there is no explanation provided for how the envelope was computed.

**Response:** Thank you for this comment. We used the fourth-order Butterworth bandpass filter and notch filter for filtering. For the envelope, we compute the local RMS value of the raw EMG via a 50-ms moving window. The manuscript was revised as follows.

The collected EMG data underwent filtering using a fourth-order Butterworth bandpass filter with cutoff frequency of 30-450 Hz and a 50 Hz notch filter [12, 13].

To determine the gait phase based on changes in the amplitude and timing of muscle activity, the envelope of an EMG signal can be obtained by computing the local RMS value of the raw EMG via a 50-ms moving window [15]. The area under the RMS envelope curve was then calculated to compare EMG activation between limbs, serving as a measure of voluntary muscle drive [16].

5. The software used for the EMG analysis is not mentioned, which is important for replicating the analysis.

**Response:** We used MATLAB 2020b for EMG analysis. We have added this information to the text.

6. Using only normative percentages to extract gait sub-cycles might not be the most accurate approach, especially given the variability among stroke patients. It might be more appropriate to use Vicon markers to manually segment the gait cycle, which could better account for individual differences in gait, particularly in stroke patients.

**Response:** Thank you for this suggestion. In this study, we used only EMG electrode which was equipped with the Vicon motion capture system to record the muscle activities. We did not use reflective markers to capture the motion. However, we used the in-shoe pressure sensor, Pedar system, which is simple and convenient, to extract the gait sub-cycle. We will consider your suggestions for our future studies.

7. As a potential future direction, as you are using Vicon you could also collect kinematic data (e.g., joint angles). This would allow you to validate claims, such as knee hyperextension, with concrete kinematic evidence.

**Response:** Thank you for this suggestion. We will consider your suggestions to use full features of Vicon system for our future studies.

Regarding figures and tables:

8. It would be better to place figure captions also with each figure, as it can be frustrating for readers to flip back and forth between the text and figures.

**Response:** Thank you for this suggestion. As we uploaded, we will put the short caption with the name of figure which might help and make it more convenient.

9. In Figure 1, it would be helpful to include abbreviation for the gait cycles in the caption for clarity.

**Response:** Thank you for this suggestion. We have added them in the caption.

**Fig 2. Gait subphase over one gait cycle which consists of first double support (DS1), single support (SS), second double support (DS2) and swing (SW) phases.**

10. The ellipsis (“…”) before (1) in Equation (1) should be removed to maintain proper formatting.

**Response:** Thank you for this comment. We have removed it.

11. From my point of view, the distinction between the meaning of RMS and RMS of the envelope is not sufficiently explained. A deeper explanation is needed to clarify the purpose of each and why one is used over the other in specific contexts.

**Response:** Thank you for this comment. We have revised the manuscript to provide a clearer explanation of the RMS and RMS of envelope as shown below.

The RMS is commonly used to quantify the overall magnitude of the EMG signal while the RMS of the envelope is used to quantify the amplitude and the timing change over gait subphases. The RMS envelope highlight muscle activity by capturing differences in timing of each subphase between limbs in both groups.

In order to determine the difference between both limbs within each group, the root mean square (RMS) was used to quantify the overall magnitude of muscle activation, as calculated using Equation (1).

$RMS= \sqrt{\frac{1}{n}\sum_{i=1}^{n} x_{i}^{2}}$	(1)

To determine the gait phase based on changes in the amplitude and timing of muscle activity, the area under the RMS envelope of each muscle was obtained in every 50 ms window of filtered EMG data [15].

12. The quality of the figures in the paper is suboptimal. Although they improve when downloaded, it would be beneficial to enhance their resolution in the manuscript itself, so that downloading is not strictly necessary.

**Response:** We apologize for this. We uploaded the high quality of image as required by the journal but when the system converted them into PDF file, it might be suboptimal quality in the manuscript.

13. The presentation of Tables 2 and 3 is confusing. You place the asterisk (*) on the right side only, which gives the impression that where are differences only on the right side, although here you are comparing both right and left. Including p-values for the comparison and marking there the significance difference between both sides would make it clearer that you are comparing between limbs.

**Response:** Thank you for your suggestion. For Table 2, we compared between left and right sides that why we put * on one column which is Right side. But for Table 3, we compared between the affected and non-affected sides. However, we would like to revise as your suggestion by state that * p<0.05 Wilcoxon signed rank test compared between limbs.

* p<0.05 Wilcoxon signed rank test compared between limbs.

14. There is also a lack of explanation for why some differences that exist in healthy subjects are absent in stroke patients, as shown when comparing Table 2 and 3. Maybe this should be discussed.

**Response:** Thank you for this comment. We have revised the manuscript to provide additional explanations in the results and discussion. The original tables were also revised for clarity: Table 2 now presents the data for healthy group, while Tables 3 and 4 presents the data for the left-affected and right-affected group of stroke patients, respectively, as shown below.

In the Results section

It clearly showed that stroke patients have greater asymmetry of lower limb muscle activity in both left-affected and right-affected groups compared to healthy subjects. The RMS values of the affected side are lower than non-affected side except in the RF and BF muscles of left-affected side group. When comparing the area under the RMS envelope curve to healthy control, stroke patients showed higher values in most muscles across gait subphases. The values were typically highest in the non-affected side, followed by the affected side, and healthy participants.

In the Discussion section

This abnormality in knee movement patterns could be a factor contributing to the poorer performance observed in the left-affected group. The higher area under RMS envelope curve values of stroke patients compared to healthy individuals possibly due to stroke disrupting normal movement coordination, which alters the timing and intensity of muscle activation in stroke patients [3, 18]. The non-affected side may compensate to ensure safer movement, for example, by increasing the activity of the GM which plays a key role in excessive hip abduction commonly observed in stroke patients with a stiff knee gait. This compensation resulting in a motion known as circumduction, which is not typically associated with the primary function of GM in hip flexion, leading to higher activity in stroke patients compared to healthy individuals [19].

15. For figures 2 and 3, you should add a legend or a caption clarifying that the shaded area represents all cycles and the line represents the mean, as this isn’t immediately obvious.

**Response:** Thank you for this comment. We have added the information for shaded areas in the captions as shown below.

Fig 3. Muscle activities of gluteus muscle, rectus femoris, biceps femoris, tibialis anterior, medial gastrocnemius, peroneus longus muscle of the left side in the healthy group across the period of (a) first double support (DS1), (b) single support (SS), (c) second double support (DS2), and (d) swing (SW) subphases over one gait cycle. Shaded area represents the range of EMG variability across participants.

Fig 4. Muscle activities of gluteus muscle, rectus femoris, biceps femoris, tibialis anterior, medial gastrocnemius, peroneus longus muscle of the right side in healthy group across the period of (a) first double support (DS1), (b) single support (SS), (c) second double support (DS2), and (d) swing (SW) subphases over one gait cycle. Shaded area represents the range of EMG variability across participants.

16. In figure 2 replace “normal person” with “healthy subject” or “healthy control” for a greater consistency and accuracy.

**Response:** Thank you for this suggestion. In the new revision, we have not put the “normal person” in the figure.

17. The footnotes for figure 6 and 7 are missing.

**Response:** Thank you for this suggestion. We have now added them to the text .

18. The orientation of the figures should be standardized (horizontal or vertical).

**Response:** Thank you for this suggestion. We have made the orientation more standardized into 2 by 2 plot.

19. In the discussion, an insufficient ankle push-off and reduced ankle plantar flexor strength were mentioned. Why was not this linked to foot drop phenomenon, a common characteristic in stroke patients?

**Response:** Thank you for this comment. We have added an explanation in the revised manuscript connecting drop foot to reduced ankle push-off and weak plantar flexor muscles, which are common in stroke patients as shown below.

Our findings reveal that the RF-MG coupling in post-stroke gait is related to abnormal coordination patterns. Such patterns include vaulting and pelvic obliquity, which may compensate for a lack of knee flexion, and circumduction, which serves as compensation in stiff knee gait [20]. Foot drop is another common disturbance during swing phase, caused by insufficient function of the TA due to impaired dorsiflexion, or from spasticity of the plantar flexion muscle [3, 18]. Altered gait patterns in stroke patients, such as stiff knee gait, can reduce foot clearance contributing to foot drop during the swing phase [3].

20. The paper mentions limitations like small sample size, but there are other areas that could benefit from further discussion:

-Variability in EMG measurements, especially given the potential differences in sensor placement and skin preparation.

- Differences in stroke severity among participants, which could influence the results.

**Response:** Thank you for this suggestion. We have added these concerns as the limitations of this study as shown below.

A larger sample size is needed to better understand the factors related to gait function in post-stroke patients. Analyzing the impact of specific brain lesions on gait performance and including a more diverse and larger patient population would provide a more comprehensive understanding of the factors influencing gait function in post-stroke individuals. Furthermore, the variability in EMG measurement (e.g., electrode placement and skin preparation) and stroke severity among participants may influence the results. It is necessary to consider these influences in future studies.

21. Conclusion

The study presents interesting and clinically relevant insights into muscle activity during walking in stroke patients compared to healthy individuals. However, several methodological details, figure presentations and analytical explanations need to be clarified or improved to strengthen the manuscript. Addressing these issues will enhance the rigor and clarity of the study, making it more impactful for both clinical and academic audiences.

**Response:** Thank you for all comments to improve this manuscript. We have considered all comments and suggestions of the reviewer and make the responses to improve the manuscript. We hope that all responses and the new revision of this manuscript will be clearer and more impactful.

Responses to reviewers

**Reviewer 2**

**General comments:**

1. In the title it is better to accent the inter-limb and intra-limb correlations, that you investigate. It is not just about muscle activity.

**Response:** Thank you for your suggestion. We have already revised the title as your suggestion to make it better understood and clearer. The revised title is shown below

Inter-lower limb and intra-lower limb muscle activity correlations during walking: A comparative study of stroke patients and healthy individuals

2. Row 20 - By examing the root mean square (RMS) values and the area under the curve of RMS envelope of EMG for the gluteus medius (GM), rectus femoris (RF), biceps femoris (BF), medial gastrocnemius (MG), and peroneus longus (PL), this research was designed to enhance our understanding of muscle coordination and its complications in stroke gait rehabilitation– specify that GM, RF, BF, MG, PL are muscles.

**Response:** Thank you for your suggestion. We have already put the “muscles” to make it clearer (line 23) as shown.

By examing the root mean square (RMS) values and the area under the curve of RMS envelope of EMG for the gluteus medius (GM), rectus femoris (RF), biceps femoris (BF), medial gastrocnemius (MG), and peroneus longus (PL) muscles, this research was designed to enhance our understanding of muscle coordination and its complications in stroke gait rehabilitation.

3. Row 25 - Stroke patients exhibited knee hyperextension, attributed to muscle weaken and compensatory mechanisms, contributing to abnormal gait patterns such as stiff knee gait. Healthy participants showed muscle weakness predominantly in the proximal muscles. – Where is the difference? RF is responsible for knee extension and is a proximal muscle in your study (for stroke patients). The two proximal muscles here are GM and RF (for healthy people). A stiff knee gait could result from spasticity, not muscle weakness.

**Response:** Thank you for your suggestion.

**Revision:** Thank you for this suggestion. It was revised to make it clearer as shown in line 27-29.

Stroke patients exhibited knee hyperextension during stance phase, primarily due to muscle weakness and compensatory mechanisms, which may coexist with stiff knee gait during swing phase. Healthy participants showed proximal muscle weakness, likely age-related.

4. Row 35 - The findings highlight the necessity of focusing on training distal muscles in stroke patients or core/proximal muscles in cases where the the ankle joint is rarely moved. – If the weaknesses are found in the proximal muscles, why people should train distal?

**Response:** Thank you for your comment. In our study, we found that stroke patients exhibited greater weakness in the distal muscle than the proximal muscles, so they need to focus on distal muscle training. Additionally, as most of patients in our study are elderly, we recommended focusing on both distal muscles and core or proximal muscles to improve weight-bearing support and enhance gait performance. Therefore, we revised to make the sentence clearer (lines 31-35).

This study provides insights into the differences in muscle coordination between healthy individuals and stroke patients during various phases of walking. The findings emphasize the importance of training distal muscles in stroke patients, particularly in elderly, while also focusing on core/proximal muscles to improve weight-bearing support and gait stability, especially in cases of limited ankle movement.

5. In the Introduction is missing a review of other scientists work in the field, for example: https://www.arquivosdeneuropsiquiatria.org/article/muscle-activity-during-gait-following-stroke/; https://www.sciencedirect.com/science/article/abs/pii/S1388245720303436; https://pmc.ncbi.nlm.nih.gov/articles/PMC7028468/; <https://www.nature.com/articles/s41597-021-008813>; <https://pmc.ncbi.nlm.nih.gov/articles/PMC7502709/>

etc…

**Response:** Thank you for your suggestion. It was revised by including a review of relevant work from your suggested references as shown below.

Recently, the study of gait in stroke patients has gained significant attention due to the significant impact on motor function associated with muscle weakness, impaired coordination, and altered muscle activation patterns, specifically affecting walking abilities and mobility [4]. Several studies compared healthy subjects and stroke patients during walking and reported that the asymmetry and variability indices of spatiotemporal parameters can indicate the gait abnormalities [5, 6].

**References**

5. Wang Y, Mukaino M, Ohtsuka K, Otaka Y, Tanikawa H, Matsuda F, et al. Gait characteristics of post-stroke hemiparetic patients with different walking speeds. International Journal of Rehabilitation Research. 2020;43(1).

6. Rozanski GM, Huntley AH, Crosby LD, Schinkel-Ivy A, Mansfield A, Patterson KK. Lower limb muscle activity underlying temporal gait asymmetry post-stroke. Clinical Neurophysiology. 2020;131(8):1848-58. doi: <https://doi.org/10.1016/j.clinph.2020.04.171>.

6. You need clearly to state the purpose of the study before the Methods section.

**Response:** Thank you for your suggestion. We have already revised to clearly state the purpose of the study as shown below.

This study aimed to fill research gaps by providing a detailed analysis of muscle activity, intra-, and inter-muscular of correlations for lower limb during the gait cycle in both healthy individuals and stroke patients. By comparing the RMS values and the area under the curve of RMS of EMG envelope across different gait phases, we sought to enhance understanding of muscle coordination and its impact on gait performance, with potential implications for stroke rehabilitation.

7. Row 66 - The participants were fifteen stroke patients, and fifteen healthy individuals recruited from Songklanagarind hospital from 30 November 2021 to 14 August 2022 – Were both group participants recruited from the hospital?

**Response:** Thank you for your question. Yes, all participants were recruited from Songklanagarind hospital. Stroke patients were recruited from the rehabilitation clinic including those who attended follow-up appointments. For healthy participants, they were hospital staff or individuals connected to staff, who joined through either posters or word-of-mouth referrals from their friends or family members working at the hospital.

8. Row 69 - Stroke patients with any history of musculoskeletal problems, other neurological conditions, lower limb arthroplasty, major falls, and fear of falling were excluded. - This sentence opposes of the mentioned previous- Stroke patients exhibited knee hyperextension, attributed to muscle weaken and compensatory mechanisms, contributing to abnormal gait patterns such as stiff knee gait. Knee hyperextension, weak muscles, compensatory mechanisms, abnormal gait patterns, and stiff knee gait are all neurological and/or musculoskeletal problems.

**Response:** Thank you for your comment. I understand your concern about contradiction. In the exclusion criteria, we aimed to exclude pre-existing unrelated to stroke that might confound the analysis of gait patterns caused specifically by stroke. There was revised by adding the examples to make it more clearly.

Stroke patients with a prior history of musculoskeletal conditions unrelated to stroke for instance acute low back pain within 6 weeks prior to participation, other neurological conditions (e.g., Parkinson’s disease, apraxia, seizure), lower limb arthroplasty, major falls, and fear of falling were excluded.

9. In Table 1 you give information about the affected side of the stroke patients. Did you assess the dexterity of all participants? It is important because you find further dissimilarities in healthy subjects too. At what recovery stage of [Brunnstrom](https://www.google.com/url?sa=t&source=web&rct=j&opi=89978449&url=https://www.physio-pedia.com/Brunnstrom_Movement_Therapy&ved=2ahUKEwin9sjGzqeJAxV3efEDHTkhCsUQFnoECBQQAQ&usg=AOvVaw3SS71sdwm73oO1wm9SkWsa) are the hemiparetic subjects – this gives reflection to the results. Have you assessed some of the following: NIHSS, Fugl-Meyer, Glasgow Coma Scale, Hunt scale, Hess scale, Barthel Index, and Functional Independence Measure?

**Response:** Thank you for your insightful comment. In our study, we assessed stroke patients using the Montreal Cognitive Assessment (MoCA) scale to evaluate cognitive function and measured manual muscle strength to characterize physical impairments. These measures were chosen to focus on key aspects relevant to our research objectives.

We did not include other assessments such as the dexterity, NIHSS, Fugl-Meyer, or Brunnstrom stages, as our primary aim was to investigate muscle activity and its inter- and intra-limb correlations, rather than a comprehensive evaluation of stroke severity or motor recovery stages. However, we acknowledge that including such measures could provide additional insights and will consider this for future studies. We have revised Table 1 by adding the MoCA score of both groups and the score of manual muscle test of stroke patient group.

|  | **Stroke patients**  **(n=15)** | **Healthy subjects**  **(n=15)** |
| --- | --- | --- |
| Age (years) | 60.87 (6.99) | 59.60 (6.32) |
| Sex (n): male/female | 11/4 | 9/6 |
| Height (m) | 1.65 (0.08) | 1.60 (0.10) |
| Weight (kg) | 67.93 (11.87) | 61.63 (11.77) |
| BMI (kg/m^2^) | 24.83 (3.51) | 23.91 (2.84) |
| Walking speed (m/s) | 0.47 (0.27) | 1.17 (0.21) |
| MoCA score (Point) | 20.85 (6.01) | 25.67 (2.41) |
| Affected side (R/L) | 6/9 | - |
| Stroke onset (years) | 4.06 (3.78) | - |
| Manual muscle test (Affected/Non-affected) |  |  |
| Hip flexor | 6.91 (1.51)/9.64 (0.81) | - |
| Hip extensor | 7.18 (1.40)/9.64 (0.81) | - |
| Knee flexor | 5.64 (2.69)/9.82 (0.60) | - |
| Knee extensor | 6.18 (2.18)/9.82 (0.60) | - |
| Ankle dorsiflexor | 3.45 (2.42)/9.64 (0.81) | - |
| Ankle plantarflexor | 3.73 (2.76)/9.64 (0.81) | - |

Values are presented as mean (SD).

10. Row 81 – remove natural – it is acquired for stroke patients.

**Response:** Thank you for your suggestion. We have deleted “natural” from the context.

Then, a 5-minute rest was provided between each set to avoid fatigue that could impact their walking pattern.

11. You can give a picture of such investigated stroke patients during examination. Better to see the attached devices and the walkway.

**Response:** Thank you for your suggestion. We have added the picture during examination as shown in Figure 1.

**Fig 1.** **Experimental setup of stroke patient with attached devices during examination.**

12. How did you choose the muscles to investigate? Do you use some worldwide recommendations for EMG examinations like the Seniam protocol?

**Response:** Thank you for pointing out. The chosen muscles were selected based on their relevance to gait and motor control in stroke patients, which can be captured by using surface electromyography (EMG). For the protocol, we followed the SENIAM protocol which provides guidelines for standardized electrode placement, ensuring consistency and comparability in EMG measurement. Therefore, we have additionally added the SENIAM protocol in the texts for EMG examination as shown below.

Subsequently, Ag/AgCl electrodes were placed at the motor point of the targeted muscles, following the specific guidelines outlined in the SENIAM protocol.

13. Row 96 - The collected EMG data underwent filtering using a 30-450 Hz bandpass filter and a 50 Hz notch filter. - How did you choose that frequency range?

**Response:** Thank you for your question. The 30-450 Hz bandpass filter was chosen based on commonly recommended frequency ranges for surface EMG signals to minimize noise while retaining relevant muscle activity data. This range aligns with the methodology used in Wei et al. and Hwang et al., which guided our selection. Additionally, the 50 Hz notch filter was applied to eliminate power line interference, as this is the standard frequency of electrical noise in our region. The previous studies were added as references of the filter we used.

The collected EMG data underwent filtering using a 30-450 Hz bandpass filter and a 50 Hz notch filter [12,13].

**Reference:**

12. Wei P, Zhang J, Wang B, Hong J. Surface Electromyography and Electroencephalogram-Based Gait Phase Recognition and Correlations Between Cortical and Locomotor Muscle in the Seven Gait Phases. Frontiers in Neuroscience. 2021;15. doi: 10.3389/fnins.2021.607905.

13. Hwang B, Oh BM, Jeon D. An Optimal Method of Training the Specific Lower Limb Muscle Group Using an Exoskeletal Robot. IEEE Transactions on Neural Systems and Rehabilitation Engineering. 2018;26(4):830-8. doi: 10.1109/TNSRE.2018.2816961.

14. Besides Spearman it is good to mention other methods for estimating the surface EMG activity of humans like IcRA, Pearson: <https://www.mdpi.com/2297-8747/29/1/8>; <https://ieeexplore.ieee.org/document/6549735>

**Response:** Thank you for your helpful feedback and references. In this study, we used Spearman correlation because it works well for analyzing non-parametric data and rank-based relationship, which fit our dataset. However, we recognize that methods like IcRA and Pearson correlation could provide additional insights, especially for parametric datasets or different ways of analyzing EMG signals.

We will look into these methods and think about using them in our future analysis.

15. Row 146 - In the stroke patient group, significant differences between the affected and non-affected sides in the RMS values were found in… - You find significant differences in RMS values between the left and right sides in healthy people, which is expected due to dexterity. So here you should compare the left and right limbs in the right affected and separately in left affected stroke patients. Furthermore, it is important to say if all participants are right-handed, left-handed, or ambidextered. In your research ‘affected side ‘includes as right, as well as the left arm. Such a comparison is unacceptable. Separate the results according to the affected side (R/L) 6/9.

**Response:** Thank you for your suggestion. We appreciate the importance of clarifying these points. We did additional analysis by looking at an affected side on the left leg and an affected side on the right separately as shown in Table 3 and Table 4.

**Table 3. Comparison of the RMS and the area under the curve of RMS envelope of EMG of left-affected side stroke patients (n=9)**

| **Parameter** | **Muscle** | **Affected side (Left)** | | | |  | **Non-affected side (Right)** | | | |
| --- | --- | --- | --- | --- | --- | --- | --- | --- | --- | --- |
|  |  | **DS1** | **SS** | **DS2** | **SW** |  | **DS1** | **SS** | **DS2** | **SW** |
| RMS value | GM | 1.38 (0.09) | 1.40 (0.08) | 1.37 (0.07) | 1.40 (0.07) |  | 1.38 (0.07) | 1.40 (0.09) | 1.38 (0.07) | 1.38 (0.07) |
|  | RF | 1.50 (0.22) | 1.51 (0.17) | 1.49 (0.18) | 1.51 (0.20) |  | 1.45 (0.10) | 1.49 (0.11) | 1.43 (0.08) | 1.43^*^ (0.09) |
|  | BF | 1.56 (0.23) | 1.58 (0.23) | 1.54 (0.22) | 1.58 (0.21) |  | 1.40^*^ (0.13) | 1.43^*^ (0.13) | 1.42^*^ (0.14) | 1.41^*^ (0.11) |
|  | MG | 1.51 (0.16) | 1.56 (0.19) | 1.51 (0.14) | 1.53 (0.13) |  | 1.54 (0.13) | 1.60 (0.14) | 1.53 (0.13) | 1.54 (0.10) |
|  | TA | 1.47 (0.11) | 1.47 (0.10) | 1.47 (0.11) | 1.51 (0.11) |  | 1.51 (0.10) | 1.58^*^ (0.15) | 1.50 (0.12) | 1.49 (0.10) |
|  | PL | 1.44 (0.10) | 1.51 (0.14) | 1.46 (0.11) | 1.51 (0.14) |  | 1.52^*^ (0.11) | 1.58 (0.14) | 1.50 (0.10) | 1.55 (0.13) |
| Area under the curve of RMS envelope of EMG | GM | 3.61 (1.44) | 4.95 (3.33) | 4.36 (2.08) | 6.76 (2.94) |  | 7.12^*^ (4.90) | 11.69^*^ (10.92) | 5.68 (4.17) | 8.44 (6.28) |
|  | RF | 6.34 (3.93) | 9.18 (6.93) | 9.07 (6.03) | 12.80 (7.15) |  | 12.52^*^ (10.61) | 16.68^*^ (11.26) | 9.78 (7.66) | 12.42 (7.18) |
|  | BF | 7.23 (5.44) | 9.85 (7.90) | 8.31 (8.21) | 14.93 (12.89) |  | 21.44^*^ (21.85) | 33.74^*^ (37.49) | 19.23^*^ (21.89) | 23.70^*^ (22.69) |
|  | MG | 10.54 (8.83) | 11.28 (8.13) | 8.99 (5.41) | 14.16 (7.30) |  | 22.89^*^ (22.09) | 40.12^*^ (40.70) | 15.79^*^ (12.45) | 23.01 (16.33) |
|  | TA | 7.32 (5.73) | 11.09 (8.41) | 10.64 (7.47) | 13.22 (7.81) |  | 21.41^*^ (15.82) | 34.67^*^ (26.68) | 17.73^*^ (11.04) | 21.60^*^ (14.58) |
|  | PL | 9.13 (7.34) | 11.62 (10.16) | 9.28 (6.30) | 14.81 (9.23) |  | 22.14^*^ (14.61) | 39.33^*^ (31.25) | 18.56^*^ (10.37) | 25.37^*^ (11.99) |

Values are presented as mean (SD).

^*^ p<0.05 Wilcoxon signed rank test compared between limbs.

**Table 4. Comparison of the RMS and the area under the curve of RMS envelope of EMG of right-affected side stroke patients (n=6)**

| **Parameter** | **Muscle** | **Affected side (Right)** | | | |  | **Non-affected side (Left)** | | | |
| --- | --- | --- | --- | --- | --- | --- | --- | --- | --- | --- |
|  |  | **DS1** | **SS** | **DS2** | **SW** |  | **DS1** | **SS** | **DS2** | **SW** |
| RMS value | GM | 1.34 (0.06) | 1.34 (0.05) | 1.34 (0.06) | 1.37 (0.06) |  | 1.37 (0.07) | 1.40^*^ (0.05) | 1.37^*^ (0.04) | 1.38 (0.07) |
|  | RF | 1.38 (0.08) | 1.42 (0.10) | 1.43 (0.12) | 1.45 (0.10) |  | 1.43^*^ (0.11) | 1.48^*^ (0.10) | 1.40 (0.10) | 1.48 (0.13) |
|  | BF | 1.44 (0.11) | 1.46 (0.11) | 1.48 (0.14) | 1.55 (0.13) |  | 1.49 (0.12) | 1.54^*^ (0.08) | 1.48 (0.11) | 1.49^*^ (0.11) |
|  | MG | 1.47 (0.17) | 1.45 (0.09) | 1.45 (0.12) | 1.52 (0.15) |  | 1.56^*^ (0.11) | 1.71^*^ (0.18) | 1.51 (0.14) | 1.56 (0.13) |
|  | TA | 1.35 (0.05) | 1.38 (0.04) | 1.35 (0.04) | 1.40 (0.07) |  | 1.44^*^ (0.08) | 1.52^*^ (0.10) | 1.42^*^ (0.07) | 1.47^*^ (0.10) |
|  | PL | 1.41 (0.18) | 1.41 (0.07) | 1.42 (0.15) | 1.47 (0.12) |  | 1.48^*^ (0.09) | 1.54^*^ (0.12) | 1.46 (0.10) | 1.53 (0.10) |
| Area under the curve of RMS envelope of EMG | GM | 4.31 (3.24) | 4.28 (1.72) | 4.53 (2.43) | 7.94 (2.69) |  | 8.09^*^ (5.98) | 12.84^*^ (7.27) | 5.98^*^ (2.50) | 7.88 (5.88) |
|  | RF | 5.83 (2.84) | 8.28 (6.68) | 7.69 (5.32) | 13.74 (6.84) |  | 10.49^*^ (5.90) | 18.07^*^ (6.64) | 9.87 (6.74) | 10.11 (4.72) |
|  | BF | 12.79 (14.43) | 15.78 (24.04) | 13.39 (14.86) | 27.99 (31.51) |  | 10.07 (4.40) | 16.60^*^ (6.19) | 8.51 (3.93) | 11.39^*^ (6.97) |
|  | MG | 5.61 (4.42) | 7.74 (9.37) | 6.43 (6.14) | 10.78 (8.04) |  | 17.14^*^ (14.77) | 33.87^*^ (21.36) | 13.61^*^ (9.26) | 15.38 (11.99) |
|  | TA | 13.18 (7.24) | 17.92 (16.49) | 18.78 (16.08) | 30.25 (20.01) |  | 19.68^*^ (11.05) | 31.06^*^ (13.29) | 14.81 (8.31) | 18.11^*^ (10.28) |
|  | PL | 8.98 (6.17) | 10.46 (8.82) | 8.93 (6.44) | 18.35 (10.21) |  | 20.64^*^ (11.57) | 37.08^*^ (16.46) | 20.00^*^ (12.35) | 20.23 (12.44) |

Values are presented as mean (SD).

^*^ p<0.05 Wilcoxon signed rank test compared between limbs.

**Response:** Furthermore, we have revised the paragraphs in the Results section regarding Table 3 and Table 4 as shown below.

In the stroke patient group, the differences between the affected and non-affected sides were considered in the left-affected and right-affected sides separately as shown in Table 3 and 4. For the left-affected group, the significant differences in the RMS values were found in the BF and PL during the DS1 phase; in the BF, TA, and PL during the SS phase; in the BF during the DS2 phase; and in the RF and BF during SW phases. For the area under the curve of RMS envelope of EMG, we observed significant differences in all six muscles during the DS1 and SS phases; in the BF, MG, TA, and PL during the DS2 phase; and in the BF, TA and PL during the SW phase.

For the right-affected group, the significant differences in the RMS values were found in the RF, MG, TA and PL during the DS1 phase; in all six muscles during the SS phase; in the GM and TA during the DS2 phase; and in the BF and TA during SW phases. For the area under the curve of RMS envelope of EMG, we observed significant differences in the GM, RF, MG, TA and PL during the DS1 phase; in all six muscles during the SS phases; in the GM, MG, and PL during the DS2 phase; and in the BF and TA during the SW phase.

It clearly showed that stroke patients have greater asymmetry of lower limb muscle activity in both left-affected and right-affected groups compared to healthy subjects. The RMS values of the affected side are lower than non-affected side except in the RF and BF muscles of left-affected side group. When comparing the area under the RMS envelope curve to healthy control, stroke patients showed higher values in most muscles across gait subphases. The values were typically highest in the non-affected side, followed by the affected side, and healthy participants.

**Response:** In the Discussion section, we have further discussed based on the results from Table 3 and Table 4 as shown below.

The results highlight differences in muscle activaion and the level of effort exerted by muscles throughout the various phases of gait cycle. In the stroke patient either left-affected or right-affected groups, the outcomes align with previous research highlighting more asymmetry between limbs which will potentially affect stability [3]. The RMS values of affected side were generally lower than non-affected side in both groups, except for the RF and BF muscles of left-affected group. The increased RMS values of these muscles may indicate spasticity, potentially contributing to the abnormality of knee movement patterns in this group [3]. According to previous studies [11, 19], poorer gait performance is associated with slower walking speeds, which aligns with our findings that the left-affected group exhibited slower walking speeds compared to right-affected group. This abnormality in knee movement patterns could be a factor contributing to the poorer performance observed in the left-affected group.

16. Figs 2,3,4, and 5 are blurred. Is that real-time gait cycle presented? Is that mean, that the phases of all participants are equal-timed?

**Response:** Thank you for your question. Figures 2,3,4, and 5 present the real-time angular velocity over one gait cycle for participants in each group. To standardize the data for comparison, the gait cycle was down sampled to 101 data points, representing 100% of the gait cycle. The thick line in the plot is the mean value as you understood. We have already improved the quality of the figures as shown below.

Fig. 3

Fig. 4

Fig. 5

Fig. 6

17. Specify in the text of figures 2,3,4, and 5 which one is for the right, and which is for the left limb.

**Response:** Thank you for your suggestion. Figures 2 and 3 represent the muscle activities of left and right limbs of healthy participants, respectively. Figures 4 and 5 show the muscle activities of the affected and non-affected side of stroke patients for figure 4,5, respectively. The captions of the figures have been revised as your suggestion. We have revised the number of figures therefore Figures 2,3,4, and 5 are Figures 3,4, 5, and 6.

18. Specify in the text of figures 6 - a, b, c, d, and 7 - a, b, c, d, what information brings. It is unclear. You do not have a presentation of the figures in the text similar to 8 and 9 in row 227.

**Response:** Thank you for pointing this out. We realized that the captions for Figures 6 and 7 were initially missing, and we have now added them to the text to provide a clear explanation of the information.

19. Row 247 – You talk about the ipsilateral (left) and contralateral (right) sides in healthy group. Termins ipsilateral and contralateral marks the body side in some kind of reference to lateral lesions. They are normally used for stroke patients. How did you decide to call the left leg ipsilateral and the right contralateral?

**Response:** Thank you for your question. The terms ipsilateral and contralateral are typically used in reference to a lesion or injury, such as in stroke patients. For the healthy group, these terms were used simply to distinguish between the left and right sides in a consistent manner across all participants, regardless of the presence of a lesion. We acknowledge that this choice of terminology might lead to confusion. We have revised the words to avoid in healthy group to ensure the clarity. The caption has been revised by replacing them with left and right sides.

**Fig 11.** **Significant inter-limb Spearman’s *r*-value between the left and right sides in healthy group** during (a) first double support phase (DS1), (b) single support phase (SS), (c) second double support phase (DS2), and (d) swing phase (SW)

20. Row 288 - safe and effective movement throughout this phase, the current study identified that the RF is crucial in controlling knee extension and stabilizing the knee joint [14]. - You do not examine other knee extensors, so you can not say that.

**Response:** Thank you for your suggestion. We did not examine other knee extensors in this study. Our analysis focused on the rectus femoris (RF) as a representative muscle due to its key role in knee extension and stabilizing during gait cycle. We have revised the statement to avoid overstating our findings as shown below.

To ensure safe and effective movement throughout this phase relies on the activity of knee extensors, with the RF playing an important role in controlling knee extension and stabilizing the knee joint [22], as observed in this study.

21. Row 302 The GM, primarily involved in hip abduction, also functions as a secondary hip rotator, crucial for stabilizing the hip and assisting the BF in controlling thigh movement for hip extension during stance. - secondary hip **internal** rotator.

**Response:** Thank you for your suggestion. We have revised that statement to “secondary hip internal rotator” as your suggestion.

The GM, primarily involved in hip abduction, also functions as a secondary internal hip rotator, crucial for stabilizing the hip and assisting the BF in controlling thigh movement for hip extension during stance.

22. Row 314 In this study, the BF exhibits a dominant function facilitating knee flexion. – Again - dominant function among which knee extensors? In this study, you investigate just BF as a knee extensor!

**Response:** Thank you for pointing this out. For the knee extensors, we investigated only the biceps femoris (BF) in this study. The term “dominant function” was intended to highlight the BF’s primary role in facilitating knee flexion, as observed from the data, rather than to compare it with other knee flexors, which were not analyzed. We have revised the statement to more accurately reflect the scope of our investigation. The context was revised by avoiding “a dominant function”, as shown below.

In this study, the BF was observed to play a key role in facilitating knee flexion.

23. Row 346 Likewise, Lim et al. found that individuals with distal muscle weakness experienced greater instability during forward and backward perturbations, whereas those with proximal muscle weakness were only unstable during backward perturbations [24]. - Citation number 24 is Horlings CGC, Küng UM, van Engelen BGM, Voermans NC, Hengstman GJD, van der Kooi AJ, et al. Balance control in patients with distal versus proximal muscle weakness. Neuroscience.479 2009;164(4):1876-86. not Lim et al. No existence of not Lim et al. at all. Please revise the references.

**Response:** Thank you for catching this mistake. The citation refers to Horling et al., not Lim et al. We appreciate your careful review, and we have corrected the context to reflect the authors of the cited references. The authors’ name was changed from “Lim” to “Horling”.

Likewise, Horling et al. found that individuals with distal muscle weakness experienced greater instability during forward and backward perturbations, whereas those with proximal muscle weakness were only unstable during backward perturbations [33].

Moreover, as we have checked again. We found that there are wrong arrangement of the context and citation we put, it’s the citation for the patient group in the line of mentioning about healthy persons. So, we rearrange it as shown below.

Previous revision

Muscle fiber loss in both type I and type II fibers is associated with aging. It has been noted that type II muscles are lost more quickly than type I fibers, aligning with the observed age-related reduction in the number of motor units and the size of type II muscle fibers, particularly in the proximal muscles [20-22]. ~~Lin et al. suggested that the muscle weakness or fatigue at the ankle has a greater impact than fatigue at the knee [23]. Likewise, Lim et al. found that individuals with distal muscle weakness experienced greater instability during forward and backward perturbations, whereas those with proximal muscle weakness were only unstable during backward perturbations [24].~~ The intra-limb findings of our study align with those observed in the earlier studies, indicating that the dominant activity in healthy participants is associated with weakness in the proximal lower limb muscles. This weakness heightens the risk of falls in older adults following muscle fatigue, as proximal muscle weakness leads to greater reliance on distal muscles during high-intensity activities [25].

In contrast to stroke participants, a predominantly moderate positive correlation was observed in the proximal muscles across all phases of the gait cycle. This may attributed to greater impairment of distal muscles compared to proximal lower limb muscles, consistent with the findings of Andrew and Bohannon [26] who observed less impairment in distal muscles, specifically, ankle dorsiflexion on the non-affected side was found to be least impaired. In our study, intense activity of the GM, BF, and TA was observed on the affected side during the DS1, DS2, and SW phases, likely due to weakness in the knee and ankle.

New revision

Muscle fiber loss in both type I and type II fibers is associated with aging. It has been noted that type II muscles are lost more quickly than type I fibers, aligning with the observed age-related reduction in the number of motor units and the size of type II muscle fibers, particularly in the proximal muscles [27-29]. The intra-limb findings of our study align with those observed in the earlier studies, indicating that the dominant activity in healthy participants is associated with weakness in the proximal lower limb muscles. This weakness heightens the risk of falls in older adults following muscle fatigue, as proximal muscle weakness leads to greater reliance on distal muscles during high-intensity activities [30].

In contrast to stroke participants, a predominantly moderate positive correlation was observed in the proximal muscles across all phases of the gait cycle. This may attributed to greater impairment of distal muscles compared to proximal lower limb muscles, consistent with the findings of Andrew and Bohannon [31] who observed that proximal muscles less impairment than distal muscles. Lin et al. suggested that the muscle weakness or fatigue at the ankle has a greater impact than fatigue at the knee [32]. Likewise, Horling et al. found that individuals with distal muscle weakness experienced greater instability during forward and backward perturbations, whereas those with proximal muscle weakness were only unstable during backward perturbations [33]. In our study, intense activity of the GM, BF, and TA was observed on the affected side during the DS1, DS2, and SW phases, likely due to weakness in the knee and ankle.

24. Row 362. No activity in the BF was observed due to stroke patients being unable to perform knee flexion. On the non-affected side, activity was observed in all six muscles. These findings are consistent with Chow et al., [27] which suggested a co-contraction of the TA, MG, and PL, typically antagonist muscles that ensure smooth ankle motion. – No activity in the BF? Being unable to perform knee extension does not mean, that no muscle action is registered. Sometimes there is muscle activation, but there is not enough power to move that limb. Does that refer to both limbs? This sentence needs to be changed. You talk about **BF** activity and after that say this correlates to Chow‘s findings about co-contraction of the **TA, MG, and PL**. Maybe a mistake appeared.

Additionally, we recognize that the mention of Chow’s findings about the TA, MG, and PL was unrelated to the discussion of BF activity.

**Response:** Thank you for pointing out. The statement regarding “No activity in BF” was an overstatement. The findings indicate a reduction in BF activity rather than complete absence. This reduction aligns with Wang et al.’s findings, as we revised in the text.

During the SS phase, activity of the GM, RF, TA, and PL was noted, possibly to prevent excessive hip drop during contralateral swing, facilitate high activity of RF due to knee hyperextension, and stabilize the ankle to support body weight [10, 22]. Reduced activity in the BF was observed in stroke patients, potentially linked to their inability to achieve effective knee flexion, which aligns with previous findings associating this impairment with altered muscle activation patterns and simplified synergies during gait [34]. On the non-affected side, altered activity was observed in all six muscles. Our findings are consistent with Chow et al., [35] which observed a co-contraction of the TA, MG, and PL, typically antagonist muscles that ensure smooth ankle motion.

25. Row 366 Our study suggests a potential therapeutic approach based on age-related weakness in proximal muscles among healthy individuals, which could heighten the risk of falls when distal muscles are intensively used, leading to increased local fatigue – Therapy is for disabled and ill patients, training is for healthy subjects.

**Response:** Thank you for pointing this out. We agree with your comment and we have revised to “training approach”.

Our study suggests a potential training approach based on age-related weakness in proximal muscles among healthy individuals, which could heighten the risk of falls when distal muscles are intensively used, leading to increased local fatigue.

26. Row 387 Stroke patients exhibited knee hyperextension, often resulting from muscle weakness and compensatory strategies, contributing to abnormal coordination patterns such as stiff knee gait – In <https://pubmed.ncbi.nlm.nih.gov/26522901/> knee hyperextension is related to motor, sensory, and proprioceptive loss; spasticity; and/or ataxia on hemiparetic gait, not muscle weakness. Show your source.

**Response:** Thank you for your suggestion. We have revised that statement to make it clearer for understanding as shown below.

Stroke patients exhibited knee hyperextension during stance often due to muscle weakness and compensatory strategies, contributing to abnormal coordination patterns and potentially coexisting with stiff knee gait during swing phase.

Responses to Editor

Also, as per my comments, I recommend that the conclusion section is made clearer.
The purpose of this study should also be made clear through explanations in the discussion section.
For example, who can it help and how?

**Response:** Thank you for your suggestion. To make it clearer, we have added information that this study is valuable for rehabilitation professionals to design rehabilitation programs for stroke patients as shown below.

For stroke patients, the observed asymmetry in muscle activation throughout all gait phases indicates impaired motor control and muscle coordination resulting from neurological damage. These insights are valuable for rehabilitation professionals, such as physiotherapists, to design rehabilitation programs for stroke patients. Rehabilitation efforts should focus on reducing muscle activity asymmetries through targeted exercises and therapies to improve balance, strength, and overall gait stability.
