# Supplementary material for: Inter-lower limb and intra-lower limb muscle activity correlations during walking: A comparative study of stroke patients and healthy individuals
Source: PLoS One. 2025 May 12;20(5):e0319955. doi: 10.1371/journal.pone.0319955 (PMC12068577; doi:10.1371/journal.pone.0319955)
Supplement: Attachment — Submitted filename: review 2.docx [file pone.0319955.s004.docx]

A lot of work has been done on the article. Most of my comments have been taken into account. The authors could highlight the advantages of Spearman over the other known methods. The manuscript can still be improved in terms of:

“This study provides insights into the differences in muscle coordination between healthy individuals and stroke patients during various phases of walking. The findings emphasize the importance of training distal muscles in stroke patients, particularly in elderly, while also focusing on core/proximal muscles to improve weight-bearing support and gait stability, especially in cases of limited ankle movement.”

Here is important to say which muscles (GM, RF, BF, MG, PL) you figure out as distal and which as proximal for clearer understanding.

“It clearly showed that stroke patients have greater asymmetry of lower limb muscle activity in both left-affected and right-affected groups compared to healthy subjects. The RMS values of the affected side are lower than non-affected side except in the RF and BF muscles of left-affected side group.”

You make too bold and generalized conclusions, keeping in mind that you find almost the same RMS values in the groups – the healthy one and the two stroke groups. The difference you talk about is observed over the data of area under the curve of RMS.

It is better to put the data of the left limb and the right limb in each table one under another.
