# Supplementary material for: Inter-lower limb and intra-lower limb muscle activity correlations during walking: A comparative study of stroke patients and healthy individuals
Source: PLoS One. 2025 May 12;20(5):e0319955. doi: 10.1371/journal.pone.0319955 (PMC12068577; doi:10.1371/journal.pone.0319955)
Supplement: Attachment — Submitted filename: Response to Reviewers r2_submitted.docx [file pone.0319955.s006.docx]

**Response to reviewers**

**Reviewer #1**

**1. Reference to SENIAM protocol:**

The authors should include a proper reference to the SENIAM protocol utilized in the study. This is an important methodological detail.

**Response:** Thank for your suggestion. A proper reference to the SENIAM protocol has been added to the revised manuscript as follow.

Subsequently, Ag/AgCl electrodes were placed at the motor point of the targeted muscles, following the specific guidelines outlined in the SENIAM protocol [12].

12. Hermens HJ, Freriks B, Disselhorst-Klug C, Rau G. Development of recommendations for SEMG sensors and sensor placement procedures. Journal of Electromyography and Kinesiology. 2000;10(5):361-74. doi: https://doi.org/10.1016/S1050-6411(00)00027-4.

**2.MATLAB mention:**

The authors state that MATLAB2020 has been added to the text, but I could not find it in the revised manuscript. Please verify and ensure that it is included where appropriate.

**Response:** Thank you for your comment. We apologize for the oversight. It has been added to the “Data processing” section. The manuscript was revised as follow.

Gait variables obtained and analyzed included ground reaction force (GRF) and muscle activity measured via EMG. All EMG data analyses were conducted using MATLAB 2020b. The collected EMG data underwent filtering using a fourth-order Butterworth bandpass filter with cutoff frequency of 30-450 Hz and a 50 Hz notch filter [12, 13]. Subsequently, the filtered EMG data were synchronized with the GRF data and interpolation was performed to estimate missing data points due to differences in sampling rates.

**3.Future considerations using Vicon:**

I believe it is essential to address the point I previously raised regarding the potential future use of Vicon systems to quantitatively validate the discussion points through kinematic data. This is crucial for strengthening the scientific rigor of the study.

**Response:** Thank you for your comment. We agree that using the Vicon system for kinematic validation in future research would strengthen the scientific rigor and have acknowledged this in the discussion section as follow.

Furthermore, the variability in EMG measurement (e.g., electrode placement and skin preparation) and stroke severity among participants may influence the results. Incorporating the Vicon systems for kinematics validation has also been highlighted as a potential approach to enhance the scientific rigor of the study. It is necessary to consider these influences in future studies.

**4.Figure captions (Figures 7 onward):**
From Figure 7 onward, the captions should clarify the abbreviations used for each muscle. This will make the figures more accessible to readers.

**Response:** Thank you for your suggestion. We have added the abbreviation to a figure captions as follow.

Fig 7. The significant intra-limb Spearman’s *r*-value from left side of healthy group in (a) first double support phase (DS1), (b) single support phase (SS), (c) second double support phase (DS2), and (d) swing phase (SW)

Abbreviations: GM, gluteus medius muscle; RF, rectus femoris muscle; BF, biceps femoris muscle; MG, medial gastrocnemius muscle; TA, tibialis anterior muscle; PL, peroneus longus muscle.

Fig 8. The significant intra-limb Spearman’s *r*-value from right side of healthy group in (a) first double support phase (DS1), (b) single support phase (SS), (c) second double support phase (DS2), and (d) swing phase (SW)

Abbreviations: GM, gluteus medius muscle; RF, rectus femoris muscle; BF, biceps femoris muscle; MG, medial gastrocnemius muscle; TA, tibialis anterior muscle; PL, peroneus longus muscle.

Fig 9. The significant intra-limb Spearman’s *r*-value from affected side of stroke group in (a) first double support phase (DS1), (b) single support phase (SS), (c) second double support phase (DS2), and (d) swing phase (SW)

Abbreviations: GM, gluteus medius muscle; RF, rectus femoris muscle; BF, biceps femoris muscle; MG, medial gastrocnemius muscle; TA, tibialis anterior muscle; PL, peroneus longus muscle.

Fig 10. The significant intra-limb Spearman’s *r*-value from non-affected side of stroke group in (a) first double support phase (DS1), (b) single support phase (SS), (c) second double support phase (DS2), and (d) swing phase (SW)

Abbreviations: GM, gluteus medius muscle; RF, rectus femoris muscle; BF, biceps femoris muscle; MG, medial gastrocnemius muscle; TA, tibialis anterior muscle; PL, peroneus longus muscle.

Fig 11. Significant inter-limb Spearman’s *r*-value between the left and right sides in healthy group during (a) first double support phase (DS1), (b) single support phase (SS), (c) second double support phase (DS2), and (d) swing phase (SW)

Abbreviations: GM, gluteus medius muscle; RF, rectus femoris muscle; BF, biceps femoris muscle; MG, medial gastrocnemius muscle; TA, tibialis anterior muscle; PL, peroneus longus muscle.

Fig 12. Significant inter-limb Spearman’s *r*-value between the affected and non-affected sides in stroke group during (a) first double support phase (DS1), (b) single support phase (SS), (c) second double support phase (DS2), and (d) swing phase (SW)

Abbreviations: GM, gluteus medius muscle; RF, rectus femoris muscle; BF, biceps femoris muscle; MG, medial gastrocnemius muscle; TA, tibialis anterior muscle; PL, peroneus longus muscle.

**5.Figure quality:**

The quality of the figures remains insufficient. When zooming in to examine details, the images become completely blurred. While the authors suggest that this issue stems from the PDF conversion tool, I recommend exploring alternative conversion programs to ensure clarity. Poor figure quality significantly hinders the understanding of the data.

**Response:** Thank you for your feedback. Figures 7-12 have been revised to ensure a resolution of 600 dpi to improve the clarity. However, we do not know the quality of these figures after they are converted into PDF file because the PDF conversion tool by the editorial manager (em) system when we submitted. Therefore, we provide the external link that the reviewer can see the original files of these figures.

<https://drive.google.com/drive/folders/1INMDX19JtrxBm9RRhfTGsB7nzLL5fALB?usp=sharing>

**6.Table 2 and additional tables:**

The presentation of Table 2 remains somewhat confusing, and now there are three separate tables for each affected side. I suggest unifying these tables if possible to improve readability. Additionally, for the healthy participants, the analysis does not consider dominance (right- or left-), which might be an important factor to address.

**Response:** Thank you for your suggestion. Tables 2-4 have been revised to present each variable for all participant groups. Table 2 now includes the RMS values for healthy, left-affected, and right-affected groups, while Table 3 provides the area under the RMS curve for the same participant groups. Additionally, the lack of consideration for the dominant side has been included in the limitations as follow.

However, this current study was constrained by the lack of information regarding the specific locations of brain lesions in stroke patients and the dominant side in both groups, which importantly impacts gait performance.

**Table 2. Comparison of the RMS value between healthy participants and stroke patients for each affected side group**

| **Subjects** | **Muscle** | **Left side** | | | |  | **Right side** | | | |
| --- | --- | --- | --- | --- | --- | --- | --- | --- | --- | --- |
|  |  | **DS1** | **SS** | **DS2** | **SW** |  | **DS1** | **SS** | **DS2** | **SW** |
| Healthy | GM | 1.38 (0.07) | 1.42 (0.07) | 1.36 (0.05) | 1.42 (0.08) |  | 1.36^*^ (0.06) | 1.42 (0.10) | 1.37 (0.06) | 1.43 (0.09) |
|  | RF | 1.37 (0.07) | 1.50 (0.13) | 1.36 (0.07) | 1.52 (0.14) |  | 1.39^*^ (0.07) | 1.50 (0.12) | 1.39^*^ (0.08) | 1.51 (0.12) |
|  | BF | 1.45 (0.11) | 1.66 (0.24) | 1.44 (0.12) | 1.56 (0.17) |  | 1.42 (0.10) | 1.61^*^ (0.19) | 1.43 (0.10) | 1.61 (0.18) |
|  | MG | 1.46 (0.13) | 1.64 (0.20) | 1.45 (0.13) | 1.62 (0.21) |  | 1.46 (0.14) | 1.64 (0.21) | 1.45 (0.12) | 1.64 (0.22) |
|  | TA | 1.44 (0.12) | 1.53 (0.15) | 1.41 (0.11) | 1.53 (0.17) |  | 1.44 (0.12) | 1.52 (0.13) | 1.42 (0.10) | 1.52 (0.16) |
|  | PL | 1.39 (0.08) | 1.54 (0.20) | 1.40 (0.12) | 1.53 (0.20) |  | 1.44^*^ (0.10) | 1.53 (0.15) | 1.42 (0.12) | 1.53 (0.19) |
| Left-affected side group | GM | 1.38 (0.09) | 1.40 (0.08) | 1.37 (0.07) | 1.40 (0.07) |  | 1.38 (0.07) | 1.40 (0.09) | 1.38 (0.07) | 1.38 (0.07) |
|  | RF | 1.50 (0.22) | 1.51 (0.17) | 1.49 (0.18) | 1.51 (0.20) |  | 1.45 (0.10) | 1.49 (0.11) | 1.43 (0.08) | 1.43^*^ (0.09) |
|  | BF | 1.56 (0.23) | 1.58 (0.23) | 1.54 (0.22) | 1.58 (0.21) |  | 1.40^*^ (0.13) | 1.43^*^ (0.13) | 1.42^*^ (0.14) | 1.41^*^ (0.11) |
|  | MG | 1.51 (0.16) | 1.56 (0.19) | 1.51 (0.14) | 1.53 (0.13) |  | 1.54 (0.13) | 1.60 (0.14) | 1.53 (0.13) | 1.54 (0.10) |
|  | TA | 1.47 (0.11) | 1.47 (0.10) | 1.47 (0.11) | 1.51 (0.11) |  | 1.51 (0.10) | 1.58^*^ (0.15) | 1.50 (0.12) | 1.49 (0.10) |
|  | PL | 1.44 (0.10) | 1.51 (0.14) | 1.46 (0.11) | 1.51 (0.14) |  | 1.52^*^ (0.11) | 1.58 (0.14) | 1.50 (0.10) | 1.55 (0.13) |
| Right-affected side group | GM | 1.37 (0.07) | 1.40 (0.05) | 1.37 (0.04) | 1.38 (0.07) |  | 1.34 (0.06) | 1.34^*^ (0.05) | 1.34^*^ (0.06) | 1.37 (0.06) |
|  | RF | 1.43 (0.11) | 1.48 (0.10) | 1.40 (0.10) | 1.48 (0.13) |  | 1.38^*^ (0.08) | 1.42^*^ (0.10) | 1.43 (0.12) | 1.45 (0.10) |
|  | BF | 1.49 (0.12) | 1.54 (0.08) | 1.48 (0.11) | 1.49 (0.11) |  | 1.44 (0.11) | 1.46^*^ (0.11) | 1.48 (0.14) | 1.55^*^ (0.13) |
|  | MG | 1.56 (0.11) | 1.71 (0.18) | 1.51 (0.14) | 1.56 (0.13) |  | 1.47^*^ (0.17) | 1.45^*^ (0.09) | 1.45 (0.12) | 1.52 (0.15) |
|  | TA | 1.44 (0.08) | 1.52 (0.10) | 1.42 (0.07) | 1.47 (0.10) |  | 1.35^*^ (0.05) | 1.38^*^ (0.04) | 1.35^*^ (0.04) | 1.40^*^ (0.07) |
|  | PL | 1.48 (0.09) | 1.54 (0.12) | 1.46 (0.10) | 1.53 (0.10) |  | 1.41^*^ (0.18) | 1.41^*^ (0.07) | 1.42 (0.15) | 1.47 (0.12) |

Values are presented as mean (SD).

^*^ p<0.05 Wilcoxon signed rank test compared between limbs in the same group.

**Table 3. Comparison of the area under the curve between RMS envelope of EMG of healthy participants and stroke patients for each affected side group**

| **Subjects** | **Muscle** | **Left side** | | | |  | **Right side** | | | |
| --- | --- | --- | --- | --- | --- | --- | --- | --- | --- | --- |
|  |  | **DS1** | **SS** | **DS2** | **SW** |  | **DS1** | **SS** | **DS2** | **SW** |
| Healthy | GM | 2.71 (1.85) | 6.40 (3.98) | 2.77 (2.24) | 6.48 (4.13) |  | 2.42 (1.06) | 5.87 (2.38) | 2.54 (1.51) | 7.01 (5.20) |
|  | RF | 2.87 (1.86) | 6.35 (3.50) | 3.14 (2.74) | 6.94 (3.94) |  | 3.04^*^ (1.88) | 6.33 (2.63) | 2.68 (1.45) | 6.76 (2.31) |
|  | BF | 5.26 (3.96) | 12.98 (8.94) | 4.77 (4.81) | 11.53 (8.68) |  | 8.28^*^ (8.05) | 18.57 (17.66) | 6.46^*^ (5.83) | 18.95^*^ (14.34) |
|  | MG | 12.05 (11.81) | 29.29 (18.96) | 11.74 (11.48) | 26.30 (23.15) |  | 11.15 (10.08) | 22.59^*^ (16.90) | 9.72^*^ (9.58) | 25.63 (24.43) |
|  | TA | 11.37 (6.88) | 24.71 (12.53) | 11.30 (6.35) | 26.10 (11.90) |  | 11.25 (6.85) | 27.60^*^ (15.33) | 13.38^*^ (8.56) | 31.71^*^ (15.80) |
|  | PL | 10.40 (6.87) | 32.27 (20.32) | 10.55 (9.50) | 23.11 (16.12) |  | 15.20^*^ (11.79) | 39.76^*^ (27.67) | 16.00^*^ (11.27) | 31.11^*^ (17.85) |
| Left-affected side group | GM | 3.61 (1.44) | 4.95 (3.33) | 4.36 (2.08) | 6.76 (2.94) |  | 7.12^*^ (4.90) | 11.69^*^ (10.92) | 5.68 (4.17) | 8.44 (6.28) |
|  | RF | 6.34 (3.93) | 9.18 (6.93) | 9.07 (6.03) | 12.80 (7.15) |  | 12.52^*^ (10.61) | 16.68^*^ (11.26) | 9.78 (7.66) | 12.42 (7.18) |
|  | BF | 7.23 (5.44) | 9.85 (7.90) | 8.31 (8.21) | 14.93 (12.89) |  | 21.44^*^ (21.85) | 33.74^*^ (37.49) | 19.23^*^ (21.89) | 23.70^*^ (22.69) |
|  | MG | 10.54 (8.83) | 11.28 (8.13) | 8.99 (5.41) | 14.16 (7.30) |  | 22.89^*^ (22.09) | 40.12^*^ (40.70) | 15.79^*^ (12.45) | 23.01 (16.33) |
|  | TA | 7.32 (5.73) | 11.09 (8.41) | 10.64 (7.47) | 13.22 (7.81) |  | 21.41^*^ (15.82) | 34.67^*^ (26.68) | 17.73^*^ (11.04) | 21.60^*^ (14.58) |
|  | PL | 9.13 (7.34) | 11.62 (10.16) | 9.28 (6.30) | 14.81 (9.23) |  | 22.14^*^ (14.61) | 39.33^*^ (31.25) | 18.56^*^ (10.37) | 25.37^*^ (11.99) |
| Right-affected side group | GM | 8.09 (5.98) | 12.84 (7.27) | 5.98 (2.50) | 7.88 (5.88) |  | 4.31^*^ (3.24) | 4.28^*^ (1.72) | 4.53^*^ (2.43) | 7.94 (2.69) |
|  | RF | 10.49 (5.90) | 18.07 (6.64) | 9.87 (6.74) | 10.11 (4.72) |  | 5.83^*^ (2.84) | 8.28^*^ (6.68) | 7.69 (5.32) | 13.74 (6.84) |
|  | BF | 10.07 (4.40) | 16.60 (6.19) | 8.51 (3.93) | 11.39 (6.97) |  | 12.79 (14.43) | 15.78^*^ (24.04) | 13.39 (14.86) | 27.99^*^ (31.51) |
|  | MG | 17.14 (14.77) | 33.87 (21.36) | 13.61 (9.26) | 15.38 (11.99) |  | 5.61^*^ (4.42) | 7.74^*^ (9.37) | 6.43^*^ (6.14) | 10.78 (8.04) |
|  | TA | 19.68 (11.05) | 31.06 (13.29) | 14.81 (8.31) | 18.11 (10.28) |  | 13.18^*^ (7.24) | 17.92^*^ (16.49) | 18.78 (16.08) | 30.25^*^ (20.01) |
|  | PL | 20.64 (11.57) | 37.08 (16.46) | 20.00 (12.35) | 20.23 (12.44) |  | 8.98^*^ (6.17) | 10.46^*^ (8.82) | 8.93^*^ (6.44) | 18.35 (10.21) |

Values are presented as mean (SD).

^*^ p<0.05 Wilcoxon signed rank test compared between limbs.

**Reviewer #2:**

1.“This study provides insights into the differences in muscle coordination between healthy individuals and stroke patients during various phases of walking. The findings emphasize the importance of training distal muscles in stroke patients, particularly in elderly, while also focusing on core/proximal muscles to improve weight-bearing support and gait stability, especially in cases of limited ankle movement.”

Here is important to say which muscles (GM, RF, BF, MG, PL) you figure out as distal and which as proximal for clearer understanding.

**Response:** Thank you for your suggestion. The abstract was revised as follow to make it clearer.

This study provides insights into the differences in muscle coordination between healthy individuals and stroke patients during various phases of walking. The findings emphasize the importance of training distal muscles, including the MG, TA, and PL muscles, in stroke patients, particularly in elderly, while also focusing on core/proximal muscles like the GM, RF, and BF muscles to improve weight-bearing support and gait stability, especially in cases of limited ankle movement.

2.“It clearly showed that stroke patients have greater asymmetry of lower limb muscle activity in both left-affected and right-affected groups compared to healthy subjects. The RMS values of the affected side are lower than non-affected side except in the RF and BF muscles of left-affected side group.”

You make too bold and generalized conclusions, keeping in mind that you find almost the same RMS values in the groups – the healthy one and the two stroke groups. The difference you talk about is observed over the data of area under the curve of RMS.

**Response:** Thank you for your suggestion. The manuscript was revised to better represent the findings as follow.

It suggests that stroke patients have greater asymmetry of lower limb muscle activity in both left-affected and right-affected groups compared to healthy subjects. While the RMS values of the affected side are lower than non-affected side, this difference is particularly evident in the area under the curve of RMS EMG data, with the exception of the RF and BF muscles of left-affected side group.

3.It is better to put the data of the left limb and the right limb in each table one under another.

**Response:** Thank you for your suggestion. Tables 2-4 have been revised to present each variable for all participant groups. Table 2 now includes the RMS values for healthy, left-affected, and right-affected groups, while Table 3 provides the area under the RMS curve for the same participant groups.

**Table 2. Comparison of the RMS value between healthy participants and stroke patients for each affected side group**

| **Subjects** | **Muscle** | **Left side** | | | |  | **Right side** | | | |
| --- | --- | --- | --- | --- | --- | --- | --- | --- | --- | --- |
|  |  | **DS1** | **SS** | **DS2** | **SW** |  | **DS1** | **SS** | **DS2** | **SW** |
| Healthy | GM | 1.38 (0.07) | 1.42 (0.07) | 1.36 (0.05) | 1.42 (0.08) |  | 1.36^*^ (0.06) | 1.42 (0.10) | 1.37 (0.06) | 1.43 (0.09) |
|  | RF | 1.37 (0.07) | 1.50 (0.13) | 1.36 (0.07) | 1.52 (0.14) |  | 1.39^*^ (0.07) | 1.50 (0.12) | 1.39^*^ (0.08) | 1.51 (0.12) |
|  | BF | 1.45 (0.11) | 1.66 (0.24) | 1.44 (0.12) | 1.56 (0.17) |  | 1.42 (0.10) | 1.61^*^ (0.19) | 1.43 (0.10) | 1.61 (0.18) |
|  | MG | 1.46 (0.13) | 1.64 (0.20) | 1.45 (0.13) | 1.62 (0.21) |  | 1.46 (0.14) | 1.64 (0.21) | 1.45 (0.12) | 1.64 (0.22) |
|  | TA | 1.44 (0.12) | 1.53 (0.15) | 1.41 (0.11) | 1.53 (0.17) |  | 1.44 (0.12) | 1.52 (0.13) | 1.42 (0.10) | 1.52 (0.16) |
|  | PL | 1.39 (0.08) | 1.54 (0.20) | 1.40 (0.12) | 1.53 (0.20) |  | 1.44^*^ (0.10) | 1.53 (0.15) | 1.42 (0.12) | 1.53 (0.19) |
| Left-affected side group | GM | 1.38 (0.09) | 1.40 (0.08) | 1.37 (0.07) | 1.40 (0.07) |  | 1.38 (0.07) | 1.40 (0.09) | 1.38 (0.07) | 1.38 (0.07) |
|  | RF | 1.50 (0.22) | 1.51 (0.17) | 1.49 (0.18) | 1.51 (0.20) |  | 1.45 (0.10) | 1.49 (0.11) | 1.43 (0.08) | 1.43^*^ (0.09) |
|  | BF | 1.56 (0.23) | 1.58 (0.23) | 1.54 (0.22) | 1.58 (0.21) |  | 1.40^*^ (0.13) | 1.43^*^ (0.13) | 1.42^*^ (0.14) | 1.41^*^ (0.11) |
|  | MG | 1.51 (0.16) | 1.56 (0.19) | 1.51 (0.14) | 1.53 (0.13) |  | 1.54 (0.13) | 1.60 (0.14) | 1.53 (0.13) | 1.54 (0.10) |
|  | TA | 1.47 (0.11) | 1.47 (0.10) | 1.47 (0.11) | 1.51 (0.11) |  | 1.51 (0.10) | 1.58^*^ (0.15) | 1.50 (0.12) | 1.49 (0.10) |
|  | PL | 1.44 (0.10) | 1.51 (0.14) | 1.46 (0.11) | 1.51 (0.14) |  | 1.52^*^ (0.11) | 1.58 (0.14) | 1.50 (0.10) | 1.55 (0.13) |
| Right-affected side group | GM | 1.37 (0.07) | 1.40 (0.05) | 1.37 (0.04) | 1.38 (0.07) |  | 1.34 (0.06) | 1.34^*^ (0.05) | 1.34^*^ (0.06) | 1.37 (0.06) |
|  | RF | 1.43 (0.11) | 1.48 (0.10) | 1.40 (0.10) | 1.48 (0.13) |  | 1.38^*^ (0.08) | 1.42^*^ (0.10) | 1.43 (0.12) | 1.45 (0.10) |
|  | BF | 1.49 (0.12) | 1.54 (0.08) | 1.48 (0.11) | 1.49 (0.11) |  | 1.44 (0.11) | 1.46^*^ (0.11) | 1.48 (0.14) | 1.55^*^ (0.13) |
|  | MG | 1.56 (0.11) | 1.71 (0.18) | 1.51 (0.14) | 1.56 (0.13) |  | 1.47^*^ (0.17) | 1.45^*^ (0.09) | 1.45 (0.12) | 1.52 (0.15) |
|  | TA | 1.44 (0.08) | 1.52 (0.10) | 1.42 (0.07) | 1.47 (0.10) |  | 1.35^*^ (0.05) | 1.38^*^ (0.04) | 1.35^*^ (0.04) | 1.40^*^ (0.07) |
|  | PL | 1.48 (0.09) | 1.54 (0.12) | 1.46 (0.10) | 1.53 (0.10) |  | 1.41^*^ (0.18) | 1.41^*^ (0.07) | 1.42 (0.15) | 1.47 (0.12) |

Values are presented as mean (SD).

^*^ p<0.05 Wilcoxon signed rank test compared between limbs in the same group.

**Table 3. Comparison of the area under the curve between RMS envelope of EMG of healthy participants and stroke patients for each affected side group**

| **Subjects** | **Muscle** | **Left side** | | | |  | **Right side** | | | |
| --- | --- | --- | --- | --- | --- | --- | --- | --- | --- | --- |
|  |  | **DS1** | **SS** | **DS2** | **SW** |  | **DS1** | **SS** | **DS2** | **SW** |
| Healthy | GM | 2.71 (1.85) | 6.40 (3.98) | 2.77 (2.24) | 6.48 (4.13) |  | 2.42 (1.06) | 5.87 (2.38) | 2.54 (1.51) | 7.01 (5.20) |
|  | RF | 2.87 (1.86) | 6.35 (3.50) | 3.14 (2.74) | 6.94 (3.94) |  | 3.04^*^ (1.88) | 6.33 (2.63) | 2.68 (1.45) | 6.76 (2.31) |
|  | BF | 5.26 (3.96) | 12.98 (8.94) | 4.77 (4.81) | 11.53 (8.68) |  | 8.28^*^ (8.05) | 18.57 (17.66) | 6.46^*^ (5.83) | 18.95^*^ (14.34) |
|  | MG | 12.05 (11.81) | 29.29 (18.96) | 11.74 (11.48) | 26.30 (23.15) |  | 11.15 (10.08) | 22.59^*^ (16.90) | 9.72^*^ (9.58) | 25.63 (24.43) |
|  | TA | 11.37 (6.88) | 24.71 (12.53) | 11.30 (6.35) | 26.10 (11.90) |  | 11.25 (6.85) | 27.60^*^ (15.33) | 13.38^*^ (8.56) | 31.71^*^ (15.80) |
|  | PL | 10.40 (6.87) | 32.27 (20.32) | 10.55 (9.50) | 23.11 (16.12) |  | 15.20^*^ (11.79) | 39.76^*^ (27.67) | 16.00^*^ (11.27) | 31.11^*^ (17.85) |
| Left-affected side group | GM | 3.61 (1.44) | 4.95 (3.33) | 4.36 (2.08) | 6.76 (2.94) |  | 7.12^*^ (4.90) | 11.69^*^ (10.92) | 5.68 (4.17) | 8.44 (6.28) |
|  | RF | 6.34 (3.93) | 9.18 (6.93) | 9.07 (6.03) | 12.80 (7.15) |  | 12.52^*^ (10.61) | 16.68^*^ (11.26) | 9.78 (7.66) | 12.42 (7.18) |
|  | BF | 7.23 (5.44) | 9.85 (7.90) | 8.31 (8.21) | 14.93 (12.89) |  | 21.44^*^ (21.85) | 33.74^*^ (37.49) | 19.23^*^ (21.89) | 23.70^*^ (22.69) |
|  | MG | 10.54 (8.83) | 11.28 (8.13) | 8.99 (5.41) | 14.16 (7.30) |  | 22.89^*^ (22.09) | 40.12^*^ (40.70) | 15.79^*^ (12.45) | 23.01 (16.33) |
|  | TA | 7.32 (5.73) | 11.09 (8.41) | 10.64 (7.47) | 13.22 (7.81) |  | 21.41^*^ (15.82) | 34.67^*^ (26.68) | 17.73^*^ (11.04) | 21.60^*^ (14.58) |
|  | PL | 9.13 (7.34) | 11.62 (10.16) | 9.28 (6.30) | 14.81 (9.23) |  | 22.14^*^ (14.61) | 39.33^*^ (31.25) | 18.56^*^ (10.37) | 25.37^*^ (11.99) |
| Right-affected side group | GM | 8.09 (5.98) | 12.84 (7.27) | 5.98 (2.50) | 7.88 (5.88) |  | 4.31^*^ (3.24) | 4.28^*^ (1.72) | 4.53^*^ (2.43) | 7.94 (2.69) |
|  | RF | 10.49 (5.90) | 18.07 (6.64) | 9.87 (6.74) | 10.11 (4.72) |  | 5.83^*^ (2.84) | 8.28^*^ (6.68) | 7.69 (5.32) | 13.74 (6.84) |
|  | BF | 10.07 (4.40) | 16.60 (6.19) | 8.51 (3.93) | 11.39 (6.97) |  | 12.79 (14.43) | 15.78^*^ (24.04) | 13.39 (14.86) | 27.99^*^ (31.51) |
|  | MG | 17.14 (14.77) | 33.87 (21.36) | 13.61 (9.26) | 15.38 (11.99) |  | 5.61^*^ (4.42) | 7.74^*^ (9.37) | 6.43^*^ (6.14) | 10.78 (8.04) |
|  | TA | 19.68 (11.05) | 31.06 (13.29) | 14.81 (8.31) | 18.11 (10.28) |  | 13.18^*^ (7.24) | 17.92^*^ (16.49) | 18.78 (16.08) | 30.25^*^ (20.01) |
|  | PL | 20.64 (11.57) | 37.08 (16.46) | 20.00 (12.35) | 20.23 (12.44) |  | 8.98^*^ (6.17) | 10.46^*^ (8.82) | 8.93^*^ (6.44) | 18.35 (10.21) |

Values are presented as mean (SD).

^*^ p<0.05 Wilcoxon signed rank test compared between limbs in the same group.
